# Communicative Interaction with and without Eye-Gaze Technology between Children and Youths with Complex Needs and Their Communication Partners

**DOI:** 10.3390/ijerph18105134

**Published:** 2021-05-12

**Authors:** Yu-Hsin Hsieh, Maria Borgestig, Deepika Gopalarao, Joy McGowan, Mats Granlund, Ai-Wen Hwang, Helena Hemmingsson

**Affiliations:** 1 Department of Special Education, Stockholm University, Se-106 91 Stockholm, Sweden; helena.hemmingsson@specped.su.se; 2 Department of Neuroscience, Uppsala University, 751 24 Uppsala, Sweden; maria.borgestig@neuro.uu.se; 3 Al Noor Training Centre for Persons with Disabilities, Building No. 01, Street No. 21 Al Barsha 1, Dubai PO 8397, United Arab Emirates; deepikarao@alnoorspneeds.ae; 4 Easterseals of Southeastern Pennsylvania, 3975 Conshohocken Ave., Philadelphia, PA 19131, USA; jmcgowan@easterseals-sepa.org; 5 CHILD, Swedish Institute of Disability Research, School of Health and Welfare, Jönköping University, 553 18 Jönköping, Sweden; mats.granlund@ju.se; 6 Graduate Institute of Early Intervention, College of Medicine, Chang-Gung University, Tao-Yuan City 33301, Taiwan; awhwang@mail.cgu.edu.tw; 7 Department of Physical Medicine and Rehabilitation, Chang Gung Memorial Hospital, Linkou, 5 Fu-Xing St., Kwei-Shan, Tao-Yuan City 33301, Taiwan

**Keywords:** complex communication needs, severe physical disabilities, eye-gaze controlled computer, communicative interaction

## Abstract

Use of eye-gaze assistive technology (EGAT) provides children/youths with severe motor and speech impairments communication opportunities by using eyes to control a communication interface on a computer. However, knowledge about how using EGAT contributes to communication and influences dyadic interaction remains limited. Aim: By video-coding dyadic interaction sequences, this study investigates the impacts of employing EGAT, compared to the Non-EGAT condition on the dyadic communicative interaction. Method: Participants were six dyads with children/youths aged 4–19 years having severe physical disabilities and complex communication needs. A total of 12 film clips of dyadic communication activities with and without EGAT in natural contexts were included. Based on a systematic coding scheme, dyadic communication behaviors were coded to determine the interactional structure and communicative functions. Data were analyzed using a three-tiered method combining group and individual analysis. Results: When using EGAT, children/youths increased initiations in communicative interactions and tended to provide more information, while communication partners made fewer communicative turns, initiations, and requests compared to the Non-EGAT condition. Communication activities, eye-control skills, and communication abilities could influence dyadic interaction. Conclusion: Use of EGAT shows potential to support communicative interaction by increasing children’s initiations and intelligibility, and facilitating symmetrical communication between dyads.

## 1. Introduction

Children and youths with complex needs, here indicating having severe physical disabilities and complex communication needs [1] such as cerebral palsy with severe motor impairments and Rett syndrome, encounter difficulties using speech for everyday communication and may be at great risk of participation restrictions in daily activities [2,3]. Without adaptations, the dysfunction of movement of children/youths with complex needs and the interplay between the impairments in cognition, motor, or other domains might hinder their communicative interaction with the environment [2,3,4]. Most often, these children use vocalization, eye pointing, facial expressions, or body movements to express needs or socialize; however, their idiosyncratic behaviors or communicative intentions may be subtle and difficult to be recognized or interpreted appropriately by those around them [5,6]. The lack of appropriate responses from communication partners and lack of communication access adapted to their motor impairments and speech difficulties could impede the children’s motivation to communicate, hinder their communication development, and restrict participation in social life [7,8,9]. Therefore, their fundamental human right of communication should be addressed [10,11].

Assistive technology (AT) as a means to enhance participation has been highlighted for children with complex needs [12]. Augmentative and alternative communication (AAC), which is one type of AT designed to facilitate communicative interactions, includes unaided methods (e.g., gestures, vocalization or speech) and aided methods (e.g., low-tech communication boards or high-tech communication devices) to supplement or compensate for the impairments of verbal communication for children with complex communication needs [13]. However, previous studies found that when aided AAC resources such as communication boards were provided, most children with complex needs required considerable assistance in using the communication interfaces and preferred the use of gesture or vocalization, with which they can communicate with familiar partners quickly and easily [5,14,15]. They occasionally used aided AAC systems via eye pointing or partner-assisted scanning when they were requested to provide information or clarification in complex conversations.

**Figure 1:**
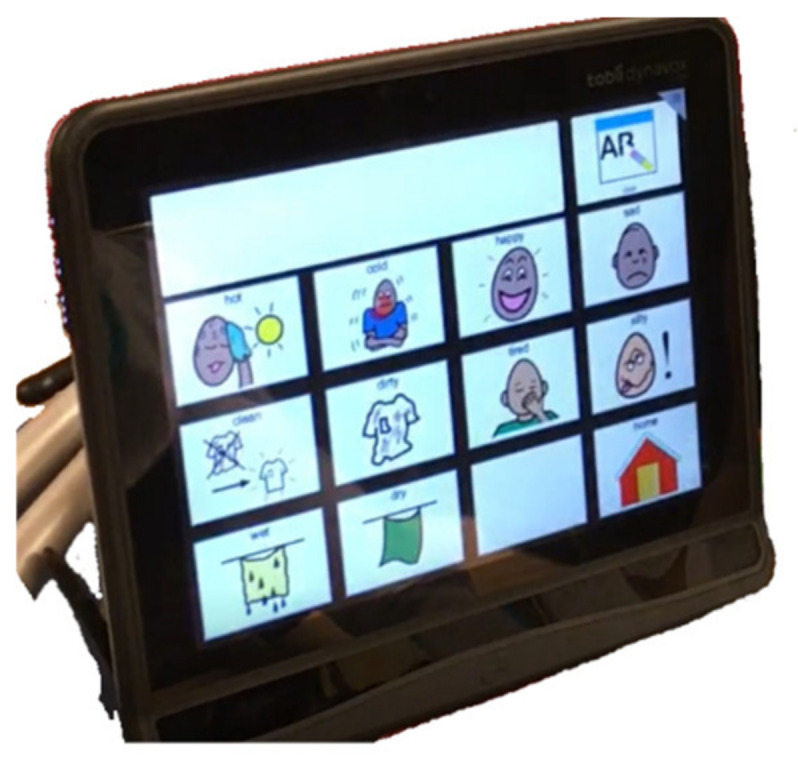
Example of EGAT with adapted communication page.

In recent years, eye-gaze assistive technology (EGAT) has been demonstrated as an opportunity for children/youths with complex needs to communicate and participate in various daily activities [16,17,18]. This innovative technology can detect eye movements using a specialized infrared video camera mounted on a tablet/computer. This calculates the direction of eye movements within a few millimeters when a child is gazing at a screen [19]. In combination with AAC software, EGAT could help children/youths with complex needs express their opinions by using their eyes to operate an AAC interface on a tablet/computer (Figure 1). It has been shown to be a feasible and relatively intuitive way to aid their communication in school or home contexts with ongoing support from communication partners and healthcare professionals [16,20,21]. Although many research studies have demonstrated communication benefits in the adult population with severe physical disabilities, there is a need for more research to support the positive effects of using EGAT in natural contexts in the children population. Some studies indicate that children with complex needs might need long-term practice to master eye-gaze control skills [22] and to develop the communicative competency to become an efficient user of communication aids [23]. Long-term practice includes both use for extended time periods and frequent use every day. Hemmingsson and Borgestig [17] reported that these children used EGAT for only a few hours per day. Besides low exposure to technology, other factors might reduce the effectiveness of EGAT, for instance, multiple impairments (e.g., visual or cognitive impairments), eye-gaze performance [22], communication abilities, learning opportunities [24], accessibility of the devices across environments [17], and attitude, knowledge and strategies of communication partners [25]. However, one recent multi-center intervention study indicated positive effects of EGAT intervention on expressive communication skills for children/youths with complex needs [26]. The stakeholders from previous studies also revealed positive experiences as the use of EGAT gave the children an opportunity to express things on their own initiative, which could lead to more opportunities to engage in communicative interaction and participate in social life [16,24].

Communicative interaction, including interactional sequences of communicative behaviors in the persons interacting (e.g., initiation of a conversation or response), communicative functions during interaction (e.g., requesting an object or answering a question), and the means of communication (e.g., using speech or eye pointing) [27], is one of the building blocks of cognitive and social development [28]. Nevertheless, research has indicated that children/youths with complex needs show a limited range of communicative interaction as they tend to play nondirective or non-initiating roles, give more adopting responses as yes–no answers, and provide less information [5,15,27]. The communication partners usually occupy more conversational space, initiate most of the interactions and request specific responses to encourage the communicative exchanges. EGAT, which requires less physical effort and assistance from communication partners than other interfaces, could provide children/youths with complex needs with opportunities to take initiative, express opinions, and interact with others [16,24]. However, few studies have provided knowledge about how use of EGAT by the children/youths influences the communicative interaction with their communication partners.

Therefore, the central aim of this study is to investigate the impacts of employing EGAT, compared to the Non-EGAT (NEGAT) condition on communicative interaction, in terms of interactional structure and communicative functions used by children/youths with complex needs and their communication partners. The research questions posed are: Which interactional structure are shown by children/youths with complex needs and their communication partners when EGAT is or is not used? Which communicative functions are used by the dyads when EGAT is or is not used? The hypotheses are: when the children/youths use EGAT in communicative interaction, (1) children/youths initiate more frequently, in contrast to the NEGAT condition; (2) communication partners take fewer communicative turns; (3) communication partners make fewer requests or demands.

The structure of this article is organized as follows. Section 2 first describes the study design, using a systematic video-coding approach to research questions and the procedure of film clip collection. Secondly, participants and the selection of film clips based on criteria are addressed. Thirdly, a coding scheme as the outcome measure for communicative interaction is detailed. Lastly, data analysis, including video analysis and the three-tiered method of analysis, is described. Section 3 presents results based on the structure of the three-tiered method. In Section 4, study findings on communicative interaction between dyads are discussed. Section 5 concludes this study.

## 2. Materials and Methods

### 2.1. Design

This study used a within-subjects design and employed a systematic video-coding approach to investigate communicative interaction when children/youths with complex needs used EGAT to interact with their communication partners in natural contexts, comparing this to the NEGAT condition. The study was part of an international multi-center EGAT intervention project, which aimed to examine the effect of a six-month EGAT intervention on participation in activities, communication, and functional independence in everyday life of children and youths with complex needs. Further details of this project can be found in the article by Borgestig et al. [26].

To confront the methodological challenges in a small population target group, a three-tiered method proposed for research on AAC use [29] was applied. The strength of this method is to combine group, intermediate and individual analysis to validate group results and provide clinically relevant information [8,29]. The application of three-tiered method for analysis was elaborated in the later Section 2.4.3.

#### Procedure

This international project collected data from the AT centers in Sweden and special needs schools in Dubai and the USA [26].

To ensure consistency of data collection, the research team in Sweden provided a checklist for the research coordinators responsible for each participating organization to collect film clips in participants’ natural contexts by the following instructions: (1) choose an activity that is meaningful and motivating for the children/youths to do, especially related to communication in natural routines (e.g., playing a game, performing an educational task, or the child communicating with the adult about something that had happened). The types of chosen activities are similar between the EGAT and NEGAT conditions; (2) choose a communication partner who know this child well; (3) film the activity about 5–10 min/day during three consecutive days in the contexts with a cell phone or a tablet on a stand; (4) choose a time of day when the children/youths would perform at their best; (5) try the best to film the faces, gaze direction and body movements of the child and his/her communication partner, and the computer screen with EGAT or low-tech communication devices.

After completion of data collection, these videos were sent to the research team in Sweden via the Cloud service, encrypted using Secure Sockets Layer.

### 2.2. Participants

#### 2.2.1. Recruitment

The original project recruited 17 children/youths with severe physical disabilities and complex communication needs and 17 of their communication partners from the organizations in three countries (nine in Sweden, five in Dubai, and three in the USA). These children/youths were candidates for and in need of EGAT after testing other aided AAC devices that had limited functional use or were unsuccessful, and they were new to EGAT before the research started.

#### 2.2.2. Selection of Participants and Film Clips

Fourteen of 17 dyads had completed video filming in their natural contexts. The first and second authors (Y-H.H., M.B.) conducted participant screening referring to the film instruction checklist. The inclusion criteria were: (1) the children/youths age was between 1 and 21 years old; (2) the language was English or Swedish; (3) at least two available videos of each participant and his/her communication partner showed they performed similar communicative interaction activities in the EGAT and NEGAT conditions (e.g., play or school learning); (4) the videos captured the dyadic facial expressions, gaze, body movements and gestures in addition to the computer screen or low-tech communication tools in order to observe their communicative interaction clearly; (5) the videos were informative, including activities generating dyadic communicative interaction instead of the child/youth only using EGAT to play games on a computer screen without interaction with the partner; (6) the videos showed the best ability of the child/youth in both the EGAT and NEGAT conditions, for instance, having more mouse clicks on screen using EGAT, showing longer attention/gaze towards the computer screen or longer interaction with partners, and/or demonstrating a high engagement level in that activity without a bored face. The exclusion criteria were (1) videos were too short (less than five minutes) and not informative; (2) videos failed to record the interactions between the dyads.

Eight dyads were not included due to language issues (*n* = 1), older age (*n* = 1), dissimilar tasks in the EGAT and NEGAT conditions (*n* = 2), or lack of communicative interaction (*n* = 4, where videos only showed the children conducting cause-effect games instead of communication activities). Based on the selection criteria, six children/youths and their communication partners were included. Dyads included three from Sweden, two from Dubai, and one from the USA. Each included dyad had four to 10 film clips of different lengths collected during the research period.

Following screening and after several meetings with two authors (M.B. and H.H.), the first author selected the best quality of two videos for each dyad, which showed similar activities, informative communicative interactions, and the best ability of children/youths in the EGAT and NEGAT conditions. A total of 12 videos from 47 film clips in six dyads were included, and each pair of videos in each dyad were concurrent or within two to five months. The videos ranged in length from five to 12 min, with a median length of eight minutes. The included videos with EGAT were recorded when the children/youths had accumulated three to six-month experiences of using EGAT, with four videos at three months of intervention and two at six months.

#### 2.2.3. Ethics Approval

The project has obtained ethical approval from the ethical review boards in Sweden (Dnr 2018/1809–32), Dubai (DSREC-11/2017_10), and the USA (protocol ESSP-02) for study implementation, data transfer and data analysis.

### 2.3. Measures

#### 2.3.1. Outcome Measures: Coding Scheme for Communicative Interaction

A coding scheme was used to investigate the characteristics of communicative interaction between the dyads, based on previous video-coding research on children with multiple disabilities and complex communication needs [5,14,15,27] with adaptations to fit the EGAT condition. The coding scheme applied to code all behaviors within the time frame of interaction included interactional structure, communicative functions, and modes of communication.

Interactional structure was classified as turns and moves [5,14]. Turns were determined by a succession of communicative signs with the boundary between turns a two-second gap supported by the presence of other behaviors, for example, non-verbal signals, pitch change, the listener took a turn, or the speaker came to a rest [14,15,27]. Each turn could include one or more moves. A move as defined by Pennington and McConachie comprised “single or strings of utterances/non-verbal communicative signals produced by one speaker within a conversational turn” [15], p.398. Moves included Initiation (I), opening the conversation or introducing a topic and could solicit a response; Response (R), a reply to an initiation; Response/Initiation (R/I), a reply to initiation but also requiring a response of its own; Follow-up (F), acknowledging the previous utterance; Follow-up/Initiation (F/I), acknowledging the previous move and requesting a response of its own; No Response. Table 1 displayed an example.

In this study, Preparatory (P) and Operation/Navigation (ON) were added to interactional moves in consideration of the preparation act to make ready for interaction (P) [5] and when the participants spend time struggling with low-tech AAC systems or EGAT with computers (ON) [23].

Communicative functions were coded to represent the intentions and purpose of the communicative act [14,15]. Each move could contain one or more codes of communicative functions [14] if the communicative purposes in the context occurred simultaneously. The categories included Requestive (RE), request for attention, information, action or clarification; Informative (IN), comments, answers, or clarification of a previous utterance; Acknowledgment (ACK), conveying understanding to previous utterance; Confirmation/Denial (CD), affirmation or rejection; Self/shared expression (SSE), demonstration of the emotional state in individuals; and Unintelligible (U), not understandable by a listener or a coder. An example was presented in Table 2.

Modes of communication were defined as the means by which communicative functions were transmitted, including speech, vocalization, gesture, and aided AAC systems [5,14,15]. The modes were combined and coded if they appeared to signal the same communicative function. Low-tech mode (Lt) was used to indicate the individual communicated using low-tech AAC, e.g., a communication book or Bliss board, and EGAT was coded to represent that persons used eye-gaze technology for aided communication.

All categories and definitions of the codes are presented in the Appendix A Table A1.

#### 2.3.2. Measures Related to Participant Characteristics

This study received participant information from the medical charts by research coordinators in the organizations. Motor severity levels were based on Gross Motor Function Classification System (GMFCS) with level I (ambulatory without restrictions) to V (limited ability to move around) [30], and Manual Ability Classification System (MACS), with level I (handles objects easily and successfully) to V (does not handle objects) [31]. Medical diagnosis, sensory functions (e.g., vision), severity of motor impairments and cognitive impairments of the children/youths were documented.

In addition, eye-control skills and communication abilities of the children/youths were assessed by Compass Aim test [32] and Communication Matrix [33], respectively, to describe the essential abilities related to use of EGAT. A trained AT specialist in the participating organization performed assessments before the EGAT intervention started.

##### Compass Aim Test

The Compass Aim test measures eye-gaze performance in computer interaction, encompassing two variables: (1) Accuracy, to measure the ability to control a mouse pointer for target selection, and (2) Time on task, to measure the required time for target selection on the screen. The test showed high test–retest reliability, good internal consistency and adequate construct validity [32].

##### Communication Matrix

The Communication Matrix details the earliest stage of communication behavior in children with severe and multiple disabilities, with seven levels, pre-intentional behavior (I), intentional behavior (II), unconventional communication (III), conventional communication (IV), concrete symbols (V), abstract symbols (VI) and language (VII). The primary level indicates the level at which the child/youth is operating predominantly. Total Percent is calculated using the total score of each item divided by the maximum possible score (= 160, each item scores from 0 to 2). The psychometrics showed good inter-observer reliability and adequate content validity [33].

### 2.4. Data Analyses

This section first described video coding analysis, followed by reliability analysis and the three-tiered method for analysis.

#### 2.4.1. Video-Coding Analysis

**Figure 2:**
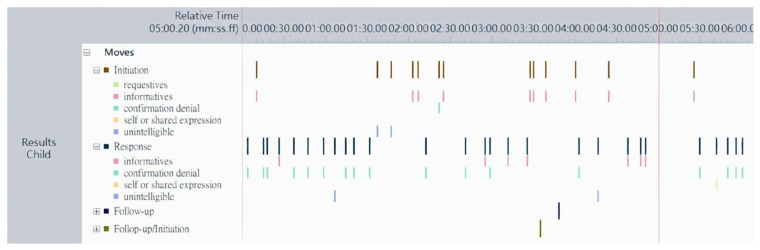
An example of video coding and visual analysis for moves and communicative functions. Note. It is a screen copy from the results of the Noldus Observer XT 14.0.

The Noldus Observer XT 14.0 (Noldus Information Technology BV, Wageningen, The Netherlands) was used in video-coding analysis, which is software for behavioral research to code and visualize behaviors on a timeline accurate to the millisecond. Figure 2 shows an example of visualization in behavioral observation of moves and communicative functions on a timeline for one participant. Each video-coding was analyzed continuously, meaning the behaviors were coded whenever they appeared and were stored in the software. The software contained statistical analyses to calculate rate per minute for each specified communicative interaction behavior in dyads. Even though the software assisted the work, it took about six to eight hours for coding and rechecking each video due to the complexity of the coding scheme and the characteristics of the target group.

#### 2.4.2. Reliability

Inter-rater reliability was determined using the kappa statistic on 10% of each video fragment (i.e., random selection of a one-minute interval) as a reliability check [34] by another rater. Due to the idiosyncratic nature of the communicative behaviors made by children/youths with complex needs, we included videos of all dyads as a reliability check since each fragment was connected with specific challenges.

The inter-rater had a background in speech-language pathology and was experienced with video-coding of communication in children with severe disabilities. Before the coding started, she received training and practiced video-coding along with the first author for 12 h online or face-to-face. Following the training, the inter-rater conducted pilot coding on two videos. Cross-examination was carried out and any discrepancies were discussed to clarify the inconsistent coding. After reaching a consensus about the definitions of each code, the inter-rater independently conducted double coding of all one-minute videos based on the coding guidelines. To avoid judgment bias due to the random selection of each video fragment, the inter-rater checked the preceding communicative behaviors before issuing a coding.

Three categories—moves, communicative functions and modes of communication—were addressed to check inter-rater reliability. Cohen’s kappa was calculated to estimate the degree of consensus between raters, according to the equation [35], in which the value above 0.75 was a good agreement, between 0.4–0.75 was an acceptable agreement, and below 0.4 was low agreement [35,36]. Inter-rater reliability revealed that the average kappa values were acceptable for moves (k = 0.72 and 0.64 in EGAT and NEGAT conditions, respectively), acceptable to good for communicative functions (k = 0.85 and 0.74), and good for modes of communication (k = 0.98 and 0.89).

#### 2.4.3. Three-Tiered Method of Analysis

To face the challenge of conducting research in this small sample and heterogeneous population and the possible occurrence of a type II error, this study utilized a three-tiered method of analysis [8,29], to firstly examine the group results of communicative interaction patterns between dyads at the general molar level, and secondly to strengthen the validity by analyzing how the communicative patterns of each dyad were congruent with or varied from the group results at the intermediate level, and lastly a further case analysis and clinical relevance at the more detailed molecular level.

Firstly, at the molar level of the group patterns, functional relationships between independent (conditions using EGAT or not) and dependent variables (communicative interaction) were examined using quantitative analysis. Rate per minute, which is the frequency divided by duration, and the proportional distribution of each communicative interaction behavior were presented using descriptive statistics (mean, standard deviation). We examined the mean differences of communicative turns in two conditions (EGAT and NEGAT) between two groups (children/youths and communication partners) using a two-way ANOVA and conducted Bonferroni post hoc analysis when the results were statistically significant. To compare the differences in frequencies of moves and communicative functions in the two conditions, parametric paired t-tests of variance was used as the data did not offend normality testing (the Shapiro–Wilk test) [37]. The significance level was set to 0.05 with two-tailed testing, and marginal significance was defined as a *p* value between 0.05 and 0.1 [38]. Data analyses were conducted using the Statistical Package for the Social Sciences (SPSS) version 25.0 (IBM Corporation, Armonk, NY, USA) and confirmed by a qualified statistician in the research team.

Secondly, at the intermediate level, the number of participants whose results were either congruent with or differed from the group result was counted to validate the group pattern. It was also possible to evaluate the degree to which individual variations influenced the mean value of the group in two conditions. In addition, a further analysis of the interrelationship between moves, communicative functions and modes of communication was conducted to determine how the dyads used their communicative functions in their initiations or response moves, and how they used EGAT and other modes, given that they were expressing specific communicative functions in the two conditions.

Lastly, at the molecular level, two cases who were typical or atypical of the group results were analyzed and compared to present the similarities and differences of the characteristics of communicative interaction and participant characteristics. It offered a basis for judgment of the potential factors from the environments and participant characteristics that could influence communicative interaction in dyads.

## 3. Results

### 3.1. Summary of Participant Characteristics, Communication Acitvities, and AAC Use

The participant characteristics are demonstrated in Table 3. The included children/youths aged from four to 19 years old (mean age: 11 years; gender female: male 5:1). All of them had severe motor impairments as level II-V in GMFCS and IV-V in MACS, with a diagnosis of cerebral palsy, Rett syndrome, or high spinal cord injury. Most of them except Molly had cognitive impairments based on medical records. Three children/youths (Laura, Peter, and Sarah) had visual impairments, with strabismus or refractive errors such as astigmatism and myopia. Their eye control skills to interact with computers showed varied abilities on accuracy (22–100%) and speed for target selection (3.42–18.25 s). Considering communication abilities, most children used unconventional communication predominantly to refuse, request, or socialize by means of facial expressions, gestures, or vocalization. They also showed varied emerging skills in using concrete symbols and conventional communication skills such as eye-pointing for expression, except for Molly, who had higher symbol-communication skills. The communication partners included three teachers, two mothers, and one occupational therapist.

As shown in Table 4, the communication activities included unstructured play activities and mealtime and structured learning tasks (e.g., letters) in school, home or hospital contexts. The children/youths used the Tobii I-series or PCEye Mini [39] as eye-gaze devices and used Communicator 5 [39], Grid 3 [40], or Communicator with WordPower [39] as AAC software in the computers. In the NEGAT condition, they were accessed to a communication board, eye-gaze frame, or iPad with different types of AAC symbols. The numbers of symbols in the AAC system varied among the children/youths, from 3 to 50 symbols per page in EGAT and from 4 to 48 symbols per page in low-tech AAC, depending on their eye-control skills and symbol-communication abilities (Table 4).

### 3.2. Group Results: Molar Level Analysis

Group results are presented in Table 5.

#### 3.2.1. Interactional Structure

Turns. The communication partners produced more communicative turns within the interaction compared to children/youths with complex needs (*F* = 13.87, *p* = 0.001). The difference in turns between the partners and children was not significant in the EGAT condition (estimated mean difference = 1.71, *p* = 0.56) but was significant in the NEGAT condition (estimated mean difference = 3.15, *p* = 0.04) from the Bonferroni analysis.

Moves. Slightly more initiations were found when children used EGAT in communication compared to the NEGAT condition (mean = 1.16 vs. 0.43, *t* = 2.26, *p* = 0.07). However, according to Table 5, R/I and F/I were not frequently found in the children, probably because of the difficulties these children had in conducting dual-purpose moves. Therefore, we collapsed I, R/I, F/I into a category representing all “initiations” because these moves all introduced a topic and/or requested responses of their own, and “initiations” were one of the most important aspects of communication for these children. Children made significantly more “initiations” when they used EGAT compared to the NEGAT condition (mean = 1.30 and 0.43, respectively, *t* = 2.60, *p* = 0.0485). In addition, the results of the analysis indicated that children made fewer response moves in the EGAT condition compared to the NEGAT condition (mean = 2.41 and 3.87, *t*= −4.76, *p* = 0.005). The partners made significantly fewer initiations (mean rate per minute = 1.97 vs. 3.87, *t*= −2.74, *p* = 0.04) in the EGAT condition compared to the NEGAT condition.

#### 3.2.2. Communicative Functions

No significant differences were found in the frequency of different categories of communicative functions used by children between the two conditions. However, of marginal significance was a finding that showed children had higher frequencies of providing information in the EGAT condition compared to the NEGAT condition (mean = 2.53 vs. 1.34, *t* = 2.04, *p* = 0.097). The partners made significantly fewer requests (mean = 3.51 vs. 5.16, *t*= −3.37, *p* = 0.02) in the EGAT condition compared to the NEGAT condition. The results also showed that they mostly dominated the conversation by asking closed-ended questions (mean = 2.56 vs. 4.26 in the EGAT and NEGAT conditions, respectively).

#### 3.2.3. Modes of Communication: EGAT and Other Modes

The analysis revealed a dominance of using EGAT as a means in communication (63%), followed by gestures (27%) for children in the EGAT condition. In the NEGAT condition, the results showed considerable variations in mode use, with gestures as the most frequent mode (48%), followed by low-tech devices in combination with gestures or vocalization (27%).

### 3.3. Analysis at the Intermediate Level

#### 3.3.1. Strength of Patterns

As seen in Table 6, most cases followed the group results, including most children making more initiations, demonstrating lower rates of response moves and higher rates of provision of information in communication in the EGAT condition compared to the NEGAT condition. In addition, the partners made fewer turns, initiations and requests in the EGAT condition compared to the NEGAT condition. However, there were a few exceptional cases. Laura demonstrated slightly lower rates of provision of information in the EGAT condition compared to the NEGAT condition (rater per minute = 1.57 vs. 1.95), which was an opposite trend from the group pattern. Her communication partner showed similar and high rates of communicative turns (rater per minute = 7.22 vs. 7.50 in the EGAT and NEGAT conditions) and made similar requests in both conditions (rater per minute = 5.83, 5.55). The other exception was Peter who showed no initiations in either condition.

In modes of communication, EGAT or conjointly with gestures and vocalization (range of proportions = 0.46–0.96) was used most by all children, and the second most commonly used mode was gestures (0.04–0.45). In the NEGAT condition, three of six children used gestures as the dominant channel (proportion = 0.53–0.73), two cases used low-tech devices conjointly with gestures or vocalization more frequently (proportion = 0.47–0.63), and one used nearly equivalent multi-methods involving gestures and vocalization.

In summary, the raw data in most cases validated the group results substantially, except the mode of communication in the NEGAT condition. A few exceptional cases existed, as seen particularly in the result of communicative interaction by Laura and her communication partner, who showed several incongruences from the group results.

#### 3.3.2. Interrelationship Analysis of Moves, Communicative Functions, and Modes of Communication

**Figure 3:**
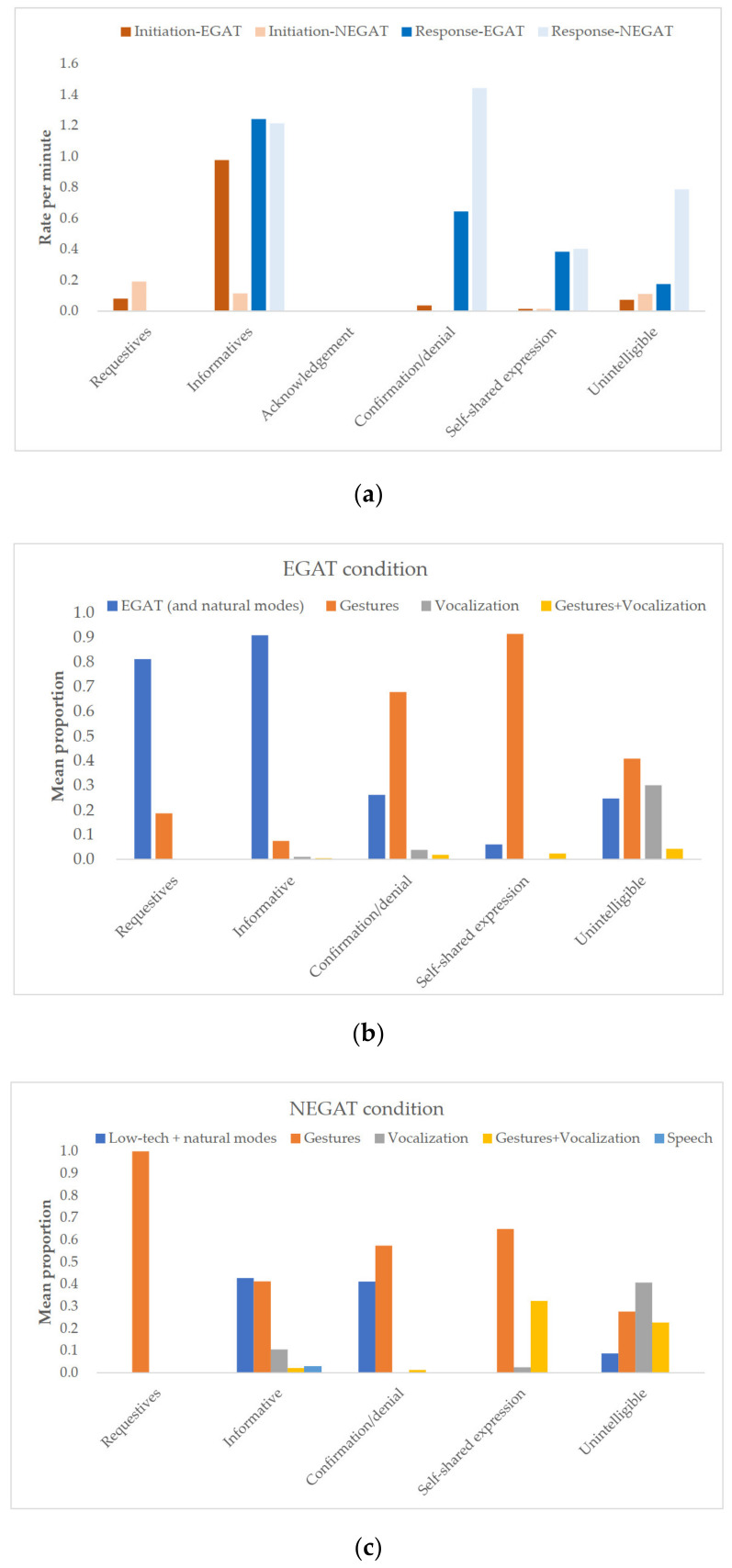
Interrelationships between moves, communicative functions and modes in children and youths with complex needs: (**a**) Interrelationships between moves (initiation and response) and communicative functions in the eye-gaze assistive technology (EGAT) and the non eye-gaze assistive technology (NEGAT) conditions; (**b**,**c**) Interrelationships between communicative functions and modes in the EGAT (**b**) and the NEGAT (**c**) conditions.

As shown in Figure 3a, in the EGAT condition, most children tended to provide provision of information in their initiations (mean rate per minute = 0.98), whereas in NEGAT condition, fewer initiations and individual variations without explicit patterns were found. In the children’s responses, provision of information was the highest communicative function in the EGAT condition (mean rate per minute = 1.24), while confirmation/denial represented the highest communicative function in the NEGAT condition (mean rate per minute = 1.44). The communication partners made almost twice the amount of requests in their initiations with higher rates in NEGAT compared to the EGAT condition (mean rate per minute = 2.84 vs.1.50).

In relation to modes, as shown in Figure 3b, the children provided information by means of EGAT primarily in their communicative turns (mean proportion = 0.94) and conveyed confirmation/denial or self-shared expression mostly by gesture (mean proportion = 0.70, 0.91). In the NEGAT condition Figure 3c, they predominantly used gestures for varied communicative functions (mean proportion = 0.57–1) except for the provision of information that they equally used gestures and a combination of low-tech devices and natural modes (mean proportion = 0.40, 0.41). The partners engaged in communicative functions predominantly by means of speech in both conditions (mean proportion = 0.56, 0.57).

### 3.4. Individual Case Studies: Analysis at the Molecular Level

One child typical of the group (Jane) and one atypical youth (Laura) who demonstrated the same diagnosis and severity of motor functions but varied in interactive patterns, were selected for further analysis. As shown in Table 6, Jane showed congruent results with most of the group patterns whereas Laura demonstrated several incongruent results.

As shown in Table 3, Jane had relatively high eye-gaze performance when using EGAT whereas Laura showed lower eye control skills (accuracy in Compass [32] = 100% vs. 36%, respectively). Jane had normal vision, and Laura had astigmatism but her performance on the Compass test was not impacted by her visual impairment, according to the report from the AT specialist. Both participants had communication abilities primarily in the level III unconventional communication [33]. However, Jane had more emerging abilities in conventional communication and concrete symbols (percent in the levels of Communication Matrix = 50%, 14.3%, respectively) compared to Laura (17.9%, 5%, respectively). Both participants used EGAT or low-tech AAC at the school or preschool.

Their film clips demonstrated different communicative interaction activities, play activities in Jane and school cognitive tasks in Laura (Table 4). In the structured school task, Laura’s teacher dominated the interaction by taking many turns in both the EGAT and NEGAT conditions (rate per minute = 7.22, 7.50), focusing on instruction and question-answer activity and requesting information, actions or clarification. Laura in turns showed quite few initiations (rate per minute = 0.37 and 0.29) but high rates of responses to answer the task (rater per minute= 1.85 and 2.82). Due to insufficient eye control skills at the early learning stage, she spent much effort operating/navigating the eye-gaze controlled computer (rate per minute = 2.04, the highest among all participants). She also showed a substantial frequency of unintelligible communicative functions particularly in the NEGAT condition (rate per min = 1.17), which might influence dyadic interaction in that the teacher would ask more questions or request clarification to receive a clear answer (see the Supplementary Materials Tables S1 and S2 for detailed data).

In summary, the individual case analysis indicated that, although Jane and Laura had similar health conditions, the form and purpose of the communication tasks in the environment (opportunities for children to take the initiative) seemed to be an essential factor for dyadic communicative interactions. Communication partners might demonstrate more directiveness in structured learning tasks than in play activities. In addition, insufficient eye control skills and communication abilities in pupils might contribute to the consequences of fewer initiations and limited information provision even when EGAT was provided.

## 4. Discussion

The central purpose of this study was to investigate the characteristics of the communicative interactions between children/youths with complex needs and their communication partners in the EGAT condition compared to the NEGAT condition. A systematic video coding was conducted to examine the interactional structure and communicative functions used by the dyads when EGAT is or is not used. The results demonstrated that when using EGAT, the children/youths initiated a conversation more frequently and tended to provide more information during the communicative interactions, while their communication partners made fewer communicative turns, initiated less frequently, and made fewer requests compared to the NEGAT condition. These findings, strengthened by a three-tiered method of analysis, indicated that when EGAT was provided, the children/youths could make a greater contribution to the communicative interaction compared to the NEGAT condition. This study supported that children as young as four could benefit from using this high technology to increase the power of communication during social interactions.

### 4.1. Initiations and Information Provision by Children in Communicative Interaction

The results of an increasing frequency of initiations in children/youths when using EGAT was encouraging. Previous video-coding research [5,14,15] showed that children with complex needs more frequently act as respondents than initiators. Their messages through body movements or gestures might be difficult to be correctly interpreted as potentially communicative by the communication partners, and their minimal movements restrict their opportunities to access many aided AAC options [5,6]. Stronger control over communicative interactions may be gained by initiating communicative interaction. Gaining more control is critical in this population in order to enjoy shared participation and build self-efficacy in communicative interaction [23]. This study demonstrated that even though these participants practiced EGAT for only three to six months, they could use it to initiate communicative interactions during play or school learning tasks. EGAT could serve as an effective method for them to gain opportunities to initiate a topic, express their opinions and increase social interactions with fewer physical demands and precisely shared referent. As a consequence, their roles as active participants could be enhanced.

The findings showed that the children/youths tended to provide information in their initiations by means of EGAT, which revealed that it could be easier for them to introduce a topic and enlarge the extent of a conversation via this AT. In contrast, in the NEGAT condition, children initiated less frequently and provided less information via gestures, vocalization or in a combination of low-tech devices. Based on the results, it is possible to inform practice that use of EGAT has the potential to provide the children/youths with better communication situations and to facilitate sharing information through age-appropriate interactions in daily life, for example, play or learning. Recent research has highlighted that the scope of communication needs to go beyond the expressions of needs and wants, and extends to development of social relationships, exchange of information and participation in social etiquette routines [42,43]. Therefore, communication partners need to facilitate access to various communication opportunities [25,44], and encourage the use of EGAT for a wider range of communicative functions in daily contexts.

### 4.2. Turns, Initiations, and Requests by Communication Partner in Communicative Interactions

As in previous studies, the result of this study revealed that communication partners showed dominance in the dyadic conversation by occupying more communicative turns, e.g., exhibiting a high frequency of initiations and requests to encourage the involvement of children in conversation in both the EGAT and NEGAT conditions. They dominated the conversation, probably as a means to scaffold and facilitate the children’s contributions according to their available repertoires [5]. Such patterns were particularly strong in structured instruction activities, for instance, as in the atypical case, Laura. However, the findings showed that in the EGAT condition, most communication partners took fewer turns and made fewer initiations and requests compared to the NEGAT condition. Because previous articles have seldom compared the communicative interactions with and without for both communicative partners and children using aided AAC, this finding adds knowledge to how eye-gaze AT could influence dyadic interactions and ameliorate the communication asymmetry. As mentioned earlier, children showed a higher frequency of initiations and information provision when using EGAT, which might reduce the burden on communicative partners in conversations. The use of EGAT may decrease the need to refer to contextual clues and prior knowledge of the children, and facilitate the interpretation of their communicative utterances [45,46]. Conversation is a bi-directional interaction, and when communication intelligibility increases in children, the efforts by communication partners to engage children in topics or clarify the content of their expression could be alleviated.

Another possible explanation for the differences seen between the conditions is that the communication partners were aware that their children were learning to use EGAT and needed time to develop their operation skills and therefore, intentionally extended the duration of waiting and encouraged children to respond or initiate their own topic using EGAT. This argument is supported by the result that more symmetrical turns in dyadic interactions were demonstrated when these children used EGAT. Since communication partners have an influence on the interactional process, their use of sensitive responding and interaction strategies (e.g., providing an expectant delay, modeling the use of communication modes, open-ended question-asking) could have positive impacts on communicative interaction for children/youths using AAC [44,47]. Further research to address the interaction strategies that communication partners use when children use EGAT or other modes could provide more insights and guide communication partner coaching on enhanced communication technologies.

### 4.3. Pros and Cons of Using Eye-Gaze AT in Communicative Interaction

This study demonstrated that EGAT could provide opportunities for children with complex needs to initiate a conversation with minimal movements and engage in a wider range of communication demands, for instance, providing explicit information to others or making comments where the natural modes of communication could not be achieved. Increasing communication intelligibility could also reduce the burden of guessing for communication partners and make the co-construction of conversation easier. This innovative technology on the children’s interactions with others could enhance a child’s control in communication and potentially increase the long-term opportunities in social participation.

Despite the advantages, the severe motor impairments in children/youths with complex needs necessitate assistance from communication partners to set up an eye-gaze controlled computer and adapt AAC content to meet their changing needs in communication [16]. Moreover, these children might take a longer time to operate/navigate computers compared with using gestures or vocalizations, particularly when they are novice users. Insufficient eye control skills at early stages of learning and cognitive demands might cause the children to tire easily which could decrease the efficiency of EGAT use over time [48]. Recent research showed children with complex needs normally used EGAT for up to two hours per day [17], which means they might use other modes of communication in communicative interactions in daily contexts more often. It is important to develop strategies and means for increasing the frequency that children are exposed to EGAT every day, i.e., embed opportunity to use EGAT in everyday activities in natural contexts, according to a newly developed clinical guideline [49]. As of today, the time spent using EGAT is too low to allow children to use the whole communicative potential.

As AAC is certainly multimodal [13], children/youths utilize multiple communication methods, by which they think fast and efficiently to fulfill communication needs depending on the communication contexts and demands. This study supported previous research [5,14,15] by showing these children demonstrated preferences using gestures or vocalizations in communicating social routines such as greetings, responding yes–no answers, or expressing affection, probably because these natural modes might be faster and more accessible for them. Thus, the findings indicated the importance of supporting their functional use of the multimodal approach in everyday contexts [50] and echoed the statement by Light and McNaughton [23] that the central focus of AAC intervention is the communication needs of children/youths with complex needs, rather than the devices.

### 4.4. Strengths and Limitations

A major strength of this study is that as far as we know, it is one of the few articles using video-coding to investigate the impacts of eye-gaze AT on communicative interaction between dyads. Using a system-based approach to coding guided by the foundation of previous research enables the meaning construction of the dyadic communicative behaviors in the EGAT and NEGAT conditions. The acceptable to good inter-rater reliability increased the credibility of this study. Higher kappa values were found in the EGAT condition compared to the NEGAT condition, indicating that the behaviors in communicative interactions were easier to observe and reach the consensus when the children/youths used EGAT compared to the other condition. Moreover, this study applied a three-tiered method, combining group, intermediate and individual analysis to validate group results and reduced the bias in that extreme cases could be hidden by group patterns [8]. Individual case studies enhanced understanding of typical and atypical individual patterns and provided a preliminary reference for further clinical implications.

Several limitations of the present study should be acknowledged. First, the small samples due to the low prevalence rates, the vulnerable health conditions [2,51], and accessibility to EGAT of our target group affect generalizability. The results should be interpreted with caution. In addition, the videos were collected from parents or teachers in order to observe their communication in natural contexts. Although instruction checklists for film clips were provided, we were finally able to use only 12 videos from six dyads for coding analysis. There were several reasons for excluding videos, for instances, technical issues, lack of information or visible behaviors in communicative interactions, or dissimilar activities in the EGAT and NEGAT conditions, which made comparisons difficult and meaningless. To reduce the likelihood of biased results from small samples, a three-tiered method was used to validate group results and provide clinically useful information. Future research could consider including trained researchers to collect film clips and, if possible, recruit more participants to consolidate the findings and shed more light on this field of knowledge. However, the time expenditure required for video-coding analysis and inter-coder training might be necessary to take into consideration.

Another limitation is that we chose the best two videos in each dyad, which could represent the communicative interactions in a specific activity. The momentary observations based on videos might not be fully representative of the dyadic communicative interactions across various daily activities. Researchers could consider a combination of different methodologies to strengthen the research findings [51], for instance, integrating interviews with proxies to triangulate quantitative data and provide a comprehensive picture of the dyadic communicative interactions in daily life.

## 5. Conclusions

The results demonstrated that the children/youths increased initiations on communicative interactions, and the communication partners decreased dominance in communicative turns and made fewer initiations in the interactional structure when using EGAT compared to the NEGAT condition. Moreover, in communicative functions, children/youths tended to provide more information using EGAT, and the communication partners made fewer requests to direct children’s responding behaviors, in contrast to the interaction in the NEGAT condition. The communication activities and the structure of the environment (e.g., play or school lessons), eye-control skills, and communication abilities could influence the dyadic interaction.

EGAT shows the potential to increase communication intelligibility, enable children/youths with complex needs gain power of communication, and facilitate the sharing of information in natural contexts. More research is needed, enrolling more participants and combining different research methods to improve generalization, and examining interactive strategies used by communication partners to support children’s communication behaviors while using EGAT to guide future interventions.

## Figures and Tables

**Table 1:** Example of moves in a play activity.

Partner: “Do you think we should put on some shoes or some pants?”	(I)
Child: “Pants” (using EGAT)	(R)
Partner: “Pants! All right. What color of pants shall we do?”	(F/I)

Note. EGAT = eye-gaze assistive technology; I = Initiation; R = Response; F/I = Follow-up/Initiation.

**Table 2:** Example of communicative functions in a play activity.

Partner: “Which color of shoes do you think we should wear?”	(RE)
Child: “Red!” (using EGAT)	(IN)
Partner: (Laugh)	(SSE)

Note. RE = Requestive; IN = Informative; SSE = Self/shared expression.

**Table 3:** Participant characteristics.

Name	Age/Sex	Diagnosis/GMFCS, MACS	Vision	Compass (Accuracy (%), Time on Task (Seconds))	Communication Matrix (Primary Level, %)	Communication Partner
Jane	6y/Female	Cerebral palsy/ V, V	Normal vision	100%, 7.41 s	Unconventional communication, 31	Teacher
Laura	16 y/Female	Cerebral palsy/ V, V	Astigmatism	36.1%, 9.76 s	Unconventional communication, 21	Teacher
Peter	19 y/Male	Cerebral palsy/ V, V	Myopia and astigmatism, with eyeglasses	22.2%, 18.25 s	Unconventional communication, 28	Teacher
Molly	4 y/Female	High spinal cord injury due to virus infection/V, V	Normal vision	100%, 3.42 s	Abstract symbols, 41	OT
Sarah	4 y/Female	Rett syndrome/ II, IV	Strabismus	33.3%, 12.44 s	Unconventional communication, 29	Mother
Anne	17 y/Female	Rett syndrome/ II, V	No vision problems with eyeglasses	39%, 12.92 s	Unconventional communication, 24	Mother

Note. GMFCS = Gross Motor Function Classification System, MACS = Manual Ability Classification System, OT = occupational therapist.

**Table 4:** Video content and use of EGAT and low-tech AAC.

Name	Condition	Video Length	Activity	Context	AAC System and Content (EGAT/Low-Tech AAC) ^1^
Jane	EGAT	7′32	Play dressing	Special preschool	EGAT: PCS symbols and photos, 12~20 symbols per page, total 140 symbols
	NEGAT	5′38	Play “finding a teacher”		Communication book: PCS symbols, pictures, colored photos, 8~20 symbols/page, total 140 symbols
Laura	EGAT	10′48	Matching/ choosing letters	Special School	EGAT: 6 PCS symbols/photos per page, total 204 symbols
	NEGAT	10′16			Eye-gaze frame: 4 single PCS symbols/colored photos per time, total < 200 symbols
Peter	EGAT	8′04	Cognitive school task	Special School	EGAT: 4 PCS symbols/photos per page, total > 120 symbols
	NEGAT	6′25			Eye-gaze frame: 4 single PCS symbols/colored photos per time, total 60 + symbols
Molly	EGAT	12′22	Pretend play using a picture book	Hospital	EGAT: Bliss symbols, 15–50 symbols/page, total 500 symbols
	NEGAT	8′09			Bliss communication board: total 540 bliss symbols. Single boards with 48 colored pictures/page
Sarah	EGAT	5′29	Meal time	Home	EGAT: PCS symbols, SymbolStix, and colored photos, 3–20 symbols/page, total 51 symbols + Sono Flex
	NEGAT	5′05			Picture pocket for 10 single symbols, LITTLE step-by-step ^2^
Anne	EGAT	7′55	Play games	Home	EGAT: Widgit symbols, colored photos, 7 symbols/page, total 62 symbols
	NEGAT	12′00			iPad: 6 symbols/page, total 100 symbols. 2–3 single colored-pictures at a time

Note. NEGAT = Non-EGAT, PCS = Picture Communication Symbols. ^1^ PCS [39], SymbolStix [39,40], and Widgit symbols [40] are different symbol sets with colored images. Little step-by step [41] is a speech-generating device to provide sequential messages with voice output. Sono Flex [39] is a communication app with about 6000 symbols in standard version. ^2^ Low tech devices received but not used in the video.

**Table 5:** Mean rate per minute (RPM) and mean proportion of turns, moves and communicative functions in the EGAT and NEGAT conditions.

Category	Children or Youths				Communication Partners			
Code	EGAT		NEGAT		EGAT		NEGAT	
	Mean RPM (SD)	Mean Proportion	Mean RPM (SD)	Mean Proportion	Mean RPM (SD)	Mean Proportion	Mean RPM (SD)	Mean Proportion
**Turns**	4.08 (1.50)	0.41	4.50 (1.68)	0.37	5.79 (1.22)	0.59	7.65 (1.91) *	0.63
**Moves**								
Preparation/Operation, Navigation	0.72 (0.80)	-	0.03 (0.08)	-	0.07 (0.08)	-	0.08 (0.12)	-
Initiation	1.16 (0.94) ^†^	0.28	0.43 (0.27)	0.10	1.97 (0.92)	0.33	3.87 (1.74) *	0.49
Response	2.41 (1.43)	0.59	3.87 (1.63) *	0.86	0.73 (0.57)	0.12	0.35 (0.21)	0.04
Response/Initiation	0.09 (0.22)	0.02	0 (0.00)	0	0.52 (0.53)	0.09	0.21 (0.19)	0.03
Follow up	0.38 (0.43)	0.09	0.19 (0.15)	0.04	1.17 (0.68)	0.19	1.24 (0.95)	0.16
Follow up/Initiation	0.04 (0.07)	0.01	0 (0.00)	0	1.62 (0.79)	0.27	2.21 (0.79)	0.28
**Communicative functions**								
Requestive	0.09 (0.08)	0.02	0.19 (0.33)	0.04	3.51 (1.41)	0.46	5.16 (1.09) *	0.54
Informative	2.53 (1.05) ^†^	0.57	1.34 (1.29)	0.29	2.09 (0.34)	0.27	2.72 (0.47)	0.28
Acknowledgement	0 (0.00)	0	0.02 (0.05)	0	1.55 (0.99)	0.20	1.19 (0.84)	0.13
Confirmation/denial	0.68 (0.92)	0.15	1.47 (2.22)	0.33	0.38 (0.39)	0.05	0.47 (0.46)	0.05
Self-shared expression	0.65 (0.90)	0.15	0.58 (0.64)	0.12	0.15 (0.15)	0.02	0.07 (0.11)	0.01
Unintelligible	0.52 (0.54)	0.12	0.98 (0.54)	0.21	0	0	0	0

RPM = rate per minute. Two-way ANOVA and Bonferroni post hoc analysis to compare the differences of communicative turns in the EGAT and the NEGAT conditions between two groups. Parametric paired t-tests to compare the differences of moves and communicative functions between two conditions in each group. * *p* < 0.05, ^†^ *p* < 0.1.

**Table 6:** Validation of group patterns by individuals at the intermediate level.

Group Patterns: Compare EGAT Condition to NEGAT Condition	Individuals					
	Jane	Laura	Peter	Molly	Sarah	Anne
Turns						
(1) Communication partners made fewer communicative turns	Yes	No	Yes	Yes	Yes	Yes
Moves						
(1) Children made more initiations	Yes	Yes	Neutral	Yes	Yes	Yes
(2) Children made fewer response moves	Yes	Yes	Yes	Yes	Yes	Yes
(3) Communication partners made fewer initiations	Yes	Yes	Yes	Yes	Yes	Yes
Communicative functions						
(1) A marginal significance that children made more provision of information	Yes	No	Yes	Yes	Yes	Yes
(2) Communication partners made fewer requests	Yes	No	Yes	Yes	Yes	Yes
Modes of communication in children and youths						
(1) In EGAT condition, a dominance of using EGAT, followed by gestures	Yes	Yes	Yes	Yes	Yes	Yes
(2) In NEGAT condition, using gestures most frequently, followed by low-tech devices in combination with gestures or vocalization	No. Low-tech with G/V, then G	No. Low-tech with G/V, then G	Yes	Yes	No. G, V, then G + V	No. G, then G + V

Yes: follow group pattern; No: deviate from group pattern; Neutral: no initiations. Abbreviations: G = Gestures, V = Vocalization.

## Data Availability

The data presented in this study are available in Supplementary Materials here.

## Supplementary Materials

The following are available online at https://www.mdpi.com/article/10.3390/ijerph18105134/s1, Table S1: Turns, moves, communicative functions and modes of communication in communication partners, Table S2: Turns, moves, communicative functions and modes of communication in children and youths with complex needs.

## Abbreviations

EGAT	Eye-gaze assistive technology
NEGAT	Non eye-gaze assistive technology
AT	Assistive technology
AAC	Augmentative and alternative communication
I	Initiation
R	Response
R/I	Response/Initiation
F/I	Follow up/Initiation
RE	Requestive
IN	Informative

## Appendix A

**Table A1:** Coding frameworks of communicative interaction and definition for each code.

Category Code	Sub-Code	Definition
Turns		A succession of communicative signs with the boundary between turns a two-second gap supported by the presence of other communicative behaviors
Moves		Comprise single or strings of utterances/non-verbal communicative signals produced by one speaker within a conversational turn
(P) Preparation		Make ready self or other person for communicative interaction
(ON) Operation/Navigation		Operate or navigate pages on computer screen using eye-gaze technology or low-tech devices
(I) Initiation		Open the conversation, introduce a topic and could solicit a response
(R) Response		Reply to an Initiation (I) or Response/Initiation (R/I)
(R/I) Response/Initiation		Reply to an I or R/I, but also require a response of its own
(F) Follow-up		Optional, acknowledge the previous utterance and require no response
(F/I) Follow-up/initiation		Acknowledge previous move and require a response of its own
Communicative functions		Coded to represent the intentions and purpose of the speaker’s communicative act
(RE) Requestive	Request joint attention	Require a listener’s attention to an object, action or the speaker
	Request information	Attempt to elicit information from a listener by using closed-ended or open questions
	Request object/action	Speaker expresses the desire for an object, activity or physical action
	Request clarification	Speaker expresses that they have not understood previous utterance and require clarification
(IN) Informative	Provision of information	Speaker makes comments about objects, actions, events, internal states, or answers to requests for information, except for confirmation/denial
	Provision of clarification	Speaker clarifies a previous utterance or turn by repetition or revision of original message
(ACK) Acknowledgement		Response or convey understanding to previous utterance or action
(CD) Confirmation-denial		Affirmation, agreement, rejection, or disagreement to yes/no questions or to the partner’s comments
(SSE) Self or shared expression		Demonstrate the speaker’s personality, or express emotional states and feelings
(U) Unintelligible		Unintelligible utterances or illocutionary force, which may not be understood by a listener or coder
Modes of communication		The manner in which communicative functions are transmitted
(S) Speech		Intelligible or unintelligible speech, which may or may not be understood by a listener or coder
(V) Vocalization		Vocal sounds not intended to be speech, but which have communicative meaning interpreted by the listener
(G) Gesture		Use eye pointing, facial expression, hand-arm gesture or body language which has illocutionary force
(Lt) low-tech AAC		Use low-tech devices, e.g., communication board by means of direct or indirect selections
(EG) EGAT		Use eye gaze assistive technology (EGAT) as a means of communication

Note. Definition for each code based on previous video-coding research [5,14,15,27].
