# Supplementary material for: Communicative Interaction with and without Eye-Gaze Technology between Children and Youths with Complex Needs and Their Communication Partners
Source: Int J Environ Res Public Health. 2021 May 12;18(10):5134. doi: 10.3390/ijerph18105134 (PMC8151590; doi:10.3390/ijerph18105134)
Supplement: Supplementary file 1 [file ijerph-18-05134-s001.zip › ijerph-1201943-supplementary.pdf]

Table S1. Turns, moves, communicative functions, and modes of communication in communication partners

Category<br>Code	Communication partners											
	Jane		Laura		Peter		Molly		Sarah		Anne	
	EGAT	NEGAT	EGAT	NEGAT	EGAT	NEGAT	EGAT	NEGAT	EGAT	NEGAT	EGAT	NEGAT
Turns (rate per minute)	7.04	10.30	7.22	7.50	4.34	5.45	6.14	9.45	5.47	7.28	4.55	5.92
Moves (rate per minute)												
Preparation	0	0	0.19	0.29	0.12	0	0	0	0	0	0.13	0.17
Initiation	2.12	4.61	3.24	4.09	1.98	2.18	2.59	6.00	0.73	4.91	1.14	1.42
Response	0.66	0.35	0.65	0.39	0.12	0	0.48	0.25	1.82	0.59	0.63	0.50
Response/Initiation	0.27	0.18	0.19	0.29	0	0	0.57	0	0.55	0.39	1.51	0.42
Follow up	2.39	3.01	0.65	1.27	1.11	1.09	0.65	1.1	1.46	0.20	0.76	0.75
Follow up/Initiation	2.25	2.48	2.59	1.27	1.61	2.65	1.86	2.57	0.91	1.18	0.50	3.08
Communicative functions (rate per minute)												
Requestives	3.98	6.03	5.83	5.55	3.34	4.67	3.64	6.62	1.64	4.32	2.65	3.75
Informatives	2.39	2.48	1.94	1.95	1.98	2.96	2.59	2.82	2.00	2.75	1.64	3.33
Acknowledgement	3.45	2.84	0.83	0.59	1.61	1.09	1.21	0.86	1.45	0.59	0.76	1.17
Confirmation/denial	0	0.18	0.74	0.97	0.99	0.93	0.24	0	0.18	0	0.13	0.75
Self-shared expression	0.27	0.18	0.09	0	0	0	0	0	0.18	0	0.38	0.25
Modes (proportion)												
Speech	0.78	0.60	0.17	0.13	0.33	0.21	0.72	0.75	0.90	0.92	0.46	0.65
Speech with eye-gaze AT	0.09	0	0.37	0	0.62	0	0.13	0	0.03	0	0.03	0
Speech with low tech devices	0	0.37	0.35	0.87	0	0.74	0	0.21	0	0	0	0.03
Speech with gestures or vocalization	0.10	0.03	0.07	0	0.05	0.03	0.13	0.01	0.03	0.07	0.41	0.26
Gestures or vocalization	0.04	0	0.04	0	0	0.03	0.01	0.03	0.03	0	0.11	0.07

[1]EGAT=eye-gaze assistive technology, NEGAT= Non-eye-gaze assistive technology

Table S2. Turns, moves, communicative functions, and modes of communication in children and youth with complex needs

Category / Code	Children and youth											
	Jane		Laura		Peter		Molly		Sarah		Anne	
	EGAT	NEGAT	EGAT	NEGAT	EGAT	NEGAT	EGAT	NEGAT	EGAT	NEGAT	EGAT	NEGAT
Turns (rate per minute)	5.71	5.68	2.22	3.41	2.97	3.58	5.95	7.12	3.47	2.56	4.17	4.67
Moves (rate per minute)												
Operation, Navigation	0.27	0	2.04	0	0.62	0	0.08	0	1.28	0.20	0	0
Initiation	0.93	0.53	0.37	0.29	0	0	1.86	0.37	1.28	0.79	2.52	0.58
Response	4.11	4.79	1.85	2.82	2.60	3.58	3.88	6.49	0.36	1.77	1.64	3.75
Response/Initiation	0	0	0	0	0	0	0	0	0.55	0	0	0
Follow up	0.66	0.35	0	0.29	0.37	0	0.16	0.25	1.09	0	0	0.25
Follow up/Initiation	0	0	0	0	0	0	0.08	0	0.18	0	0	0
Communicative functions (rate per minute)												
Requestives	0.13	0.35	0.09	0	0	0	0.16	0	0.18	0.79	0	0
Informatives	2.92	0	1.57	1.95	1.11	1.09	2.59	1.59	2.92	0	4.04	3.41
Confirmation/denial	1.46	4.25	0	0	0.12	0.16	2.18	4.41	0.18	0	0.13	0
Self-shared expression	1.19	0	0.09	0.39	2.23	1.56	0.40	0.37	0	0	0	1.17
Unintelligible	0.13	1.06	0.46	1.17	1.36	1.09	0.97	0.61	0.18	1.77	0	0.17
Modes (proportion)												
Eye-gaze AT (and natural modes[2])	0.69	0	0.85	0	0.56	0	0.46	0	0.96	0	0.73	0
Low-tech devices and natural modes[2]	0	0.47	0	0.66	0	0.26	0	0.24	0	0	0	0.04
Gestures	0.31	0.38	0.15	0.17	0.45	0.70	0.37	0.53	0.04	0.27	0.27	0.73
Vocalization	0	0.13	0	0.03	0	0	0.15	0.19	0	0.40	0	0.02
Gestures + Vocalization	0	0.03	0	0.14	0	0.04	0.03	0.02	0	0.33	0	0.22
Speech	0	0	0	0	0	0	0	0.02	0	0	0	0

[1]EGAT=eye-gaze assistive technology, NEGAT= Non-eye-gaze assistive technology. [2] Natural modes here indicate using gestures or vocalization.
